# A long journey to treat epilepsy with the gut microbiota

**DOI:** 10.3389/fncel.2024.1386205

**Published:** 2024-06-26

**Authors:** Qinrui Li, Youyu Gu, Jingjing Liang, Zhixian Yang, Jiong Qin

**Affiliations:** ^1^Department of Pediatrics, Peking University People’s Hospital, Beijing, China; ^2^Epilepsy Center, Peking University People’s Hospital, Beijing, China

**Keywords:** epilepsy, gut microbiota, brain–gut axis, probiotics, ketogenic diet

## Abstract

Epilepsy is a common neurological disorder that affects approximately 10.5 million children worldwide. Approximately 33% of affected patients exhibit resistance to all available antiseizure medications, but the underlying mechanisms are unknown and there is no effective treatment. Increasing evidence has shown that an abnormal gut microbiota may be associated with epilepsy. The gut microbiota can influence the function of the brain through multiple pathways, including the neuroendocrine, neuroimmune, and autonomic nervous systems. This review discusses the interactions between the central nervous system and the gastrointestinal tract (the brain–gut axis) and the role of the gut microbiota in the pathogenesis of epilepsy. However, the exact gut microbiota involved in epileptogenesis is unknown, and no consistent results have been obtained based on current research. Moreover, the target that should be further explored to identify a novel antiseizure drug is unclear. The role of the gut microbiota in epilepsy will most likely be uncovered with the development of genomics technology.

## 1 Introduction

Epilepsy is a chronic disease of the central nervous system (CNS) and is characterized by recurrent and unprovoked seizures (Fisher et al., 2017). Epilepsy affects more than 50–70 million people worldwide, including 10.5 million children under 15 years of age who have active epilepsy (Thijs et al., 2019; Singh and Sander, 2020). Approximately 80% of patients with epilepsy live in low- and middle-income countries (Saxena and Li, 2017; Singh and Sander, 2020). The majority of these countries are in Africa, with Uganda being affected the most(Newton and Garcia, 2012). A systematic review reported that the prevalence of epilepsy in children and young adults is approximately two-fold greater than that in middle-aged patients (Benamer and Grosset, 2009). Approximately 33% of patients suffer from refractory epilepsy, which places a tremendous burden on global health and finances (Dalic and Cook, 2016). Therefore, additional and more effective antiseizure medications are urgently needed. Many studies have shown that the gut microbiota is associated with epilepsy (Xie et al., 2017; Peng et al., 2018, 2023; Zhang et al., 2018; Huang et al., 2019, 2022; Lindefeldt et al., 2019; Gong et al., 2020, 2022; Lee et al., 2020, 2021; Safak et al., 2020; Cui et al., 2021; Xu et al., 2021; Bertuccioli et al., 2022; Dahlin et al., 2022; Dai et al., 2022; Ouyang et al., 2022; Zhou et al., 2022, 2023; Turay et al., 2023; Wan et al., 2024); however, it is difficult to determine whether the gut microbiota is the cause of epilepsy or whether changes in the microbiota are only a result of epilepsy. Therefore, a long journey lies ahead in discovering drugs that target the gut microbiota for epilepsy treatment.

The human gut hosts approximately 100 trillion microorganisms, which are collectively referred to as the gut microbiota (Saad et al., 2016). The gut microbiota defines the genes and genomes of the microbiota, as well as the products of the microbiota and the host environment (Whiteside et al., 2015). However, the association between the microbiota and heredity has not been determined. Acquired factors, such as diet and drugs, have a considerable influence on the composition of the gut microbiota (Rothschild et al., 2018). Additionally, the gut microbiota fluctuates over the lifetime of a person and changes rapidly before 3 years of age, especially during the weaning period (Ling et al., 2022).

In the human gastrointestinal tract, the most dominant bacterial phyla are *Firmicutes, Bacteroidetes, Actinobacteria, Proteobacteria, Fusobacteria, Verrucomicrobia*, and *Cyanobacteria*; among which, *Bacteroidetes* and *Firmicutes* constitute >90% of the total bacteria (Ding et al., 2021). The gut microbiota regulates many physiological functions, including digestion (El et al., 2014), absorption (Zeng et al., 2017), excretion (Karl et al., 2017), vitamin synthesis, and immune function (Cheng et al., 2019). Gastrointestinal symptoms, such as abdominal pain or diarrhea, might be the first or only obvious symptoms in individuals with epilepsy and can sometimes even lead to incorrect diagnoses in clinical practice (Ozkara et al., 2009; Yunus et al., 2016). Additionally, patients with inflammatory bowel disease have a greater risk of suffering from epilepsy (De Caro et al., 2019). According to these clinical findings, the role of the microbiota and the gut–brain axis in epilepsy should be noted. This article focuses on the gut microbiota and reviews the relationship between the gut microbiota and epilepsy and the underlying mechanisms involved. In addition, this review describes the shortcomings and limitations of related studies, such as methodology and confounding factors, and discusses the challenges and limitations of epilepsy treatment from the perspective of the intestinal flora.

## 2 The potential relationship between the gut microbiota and epilepsy

Recently, a new concept named the “brain–gut axis,” which refers to the gut microbiota, was proposed to influence the CNS (Quigley, 2017). Millions of neurons are present in the gastrointestinal mucosa. These cells constitute the enteric nervous system (ENS), regulate gastrointestinal functions, and influence the CNS mainly via the vagus nerve. Thus, the gut is considered a “second brain.” The gut microbiota acts as an important medium in the bidirectional system of communication between the brain and the gut (Quigley, 2017). This communication needs to occur through multiple systems, including the neuroendocrine, neuroimmune, and autonomic nervous systems (Martin et al., 2018). Because of the brain–gut axis, abnormal gut microbiota may disrupt the homeostasis of the CNS and may be involved in the pathogenesis of Alzheimer’s disease (Shen and Ji, 2019), Parkinson’s disease in humans and animals (Scheperjans et al., 2015; Sampson et al., 2016; Pietrucci et al., 2019; Zhang et al., 2023), stroke in humans and mice (Winek et al., 2016; Meng et al., 2023; Chen et al., 2024), autism spectrum disorder (ASD) in humans and animals (Li et al., 2017; Kong et al., 2021), and epilepsy in humans and animals (Olson et al., 2018). The oral microbiota has also been reported to be altered during epilepsy (Lian et al., 2023). Moreover, a ketogenic diet (KD) is a non-pharmacological therapy for drug-resistant epileptic patients and has good curative effects (van der Louw et al., 2016; Imdad et al., 2022). Additionally, the intestinal microbiota of infants is different from that of older children. Therefore, infants may not have generalized tonic–clonic seizures (Korff and Nordli, 2005). Here, we discuss the prototypical relationships between the gut microbiota and epilepsy through several systems.

In human studies, it is difficult to control confounding factors, such as diet, habitat, living environment, and life stress, in gut microbiota. Therefore, the results from human studies are at risk of being inconsistent. First, the α diversity of the gut microbiota in epileptic patients is not consistent. Three studies showed that the microbiota diversity of patients with epilepsy was lower than that of people without epilepsy (Xie et al., 2017; Gong et al., 2020; Cui et al., 2021); however, Peng et al. reported that the diversity of the microbiota was significantly greater in patients with drug-resistant epilepsy than in patients with drug-sensitive epilepsy and healthy controls (Peng et al., 2018). Second, the gut microbiota is altered in epileptic patients, but the results are inconsistent. Most studies have shown that epilepsy patients have an increased abundance of the phylum *Firmicutes* and a decreased abundance of the phylum *Bacteroidetes* (Xie et al., 2017; Peng et al., 2018; Lee et al., 2020). In contrast, Zhou et al. reported that *Actinobacteria*, *Bacteroidetes*, and *Proteobacteria* increased and *Firmicutes* decreased in adults with epilepsy (Zhou et al., 2023). For instance, studies on the gut microbiota in epileptic patients have yielded different results. A study showed that the abundances of the genera *Campylobacter*, *Delftia*, *Haemophilus*, *Lautropia*, and *Neisseria* among the *Proteobacteria* phylum were significantly greater in patients with epilepsy than in people without epilepsy (Safak et al., 2020). The *Fusobacteria* phylum could be detected in only 10.6% of patients with epilepsy (Safak et al., 2020). In addition, compared to those in the control group, the bacteria in the epilepsy group had increased *Proteobacteria* and *Actinobacteria* and decreased *Bacteroidetes*. Moreover, the bacteria in the epilepsy group were enriched in *Faecalibacterium, Escherichia-Shigella, Subdoligranulum*, and *unclassified Enterobacteriaceae* and decreased in *Bacteroides, Megamonas, Prevotella, unclassified Lachnospiraceae*, *and Blautia* (Cui et al., 2021). Dahlin et al. reported that the levels of *Bifidobacteria* increased after KD treatment and were associated with an antiepileptic response (Dahlin et al., 2022). These patients had structural, genetic, and unknown information, but the relationships between the etiology and the gut microbiota were not compared (Dahlin et al., 2022). *Proteobacteria* and *Cronobacter* predominate in refractory epileptic infants of unknown cause (Xie et al., 2017). Furthermore, patients with drug-resistant epilepsy had increased levels of the bacterial taxa *Actinobacteria*, *Verrucomicrobia*, and *Nitrospirae* and the genera *Blautia*, *Bifidobacterium*, *Subdoligranulum*, *Dialister*, and *Anaerostipes* (Gong et al., 2020). In children with intractable epilepsy, *Bacteroidetes* levels were lower and *Actinobacteria* levels were higher. The ATP-binding cassette (ABC) transporter-associated microbiota included the *Enterococcus faecium* group, the *Bifidobacterium longum* group, and the *Eggerthella lenta*, which are likely biomarkers of intractable epilepsy (Lee et al., 2020). However, in another study by Lee et al., the abundances of *Bacteroides finegoldii* and *Ruminococcus_g2* increased in the drug-responsive group, and the relative abundance of *Negativicutes* (belonging to *Firmicutes*) increased in the drug-resistant group (Lee et al., 2021). After KD therapy, seizures decreased significantly, electroencephalogram improved, α-diversity and the abundance of *Firmicutes* decreased, and the abundance of *Bacteroidetes* increased. In addition, *Clostridiales*, *Ruminococcaceae*, *Rikenellaceae*, *Lachnospiraceae*, and *Alistipes* were likely non-responsive patient biomarkers (Zhang et al., 2018). Compared with patients who have more than four seizures per year, patients with fewer than four seizures per year had an increase in the abundance of *Bifidobacteria* and *Lactobacillus* (Peng et al., 2018). Additionally, antibiotic treatment also reduced seizure frequency in some patients (Ghanizadeh and Berk, 2015; Cheraghmakani et al., 2021). Mu et al. also showed that manipulating the gut microbiota attenuated seizures in an animal model of infantile spasm syndrome by KD (Mu et al., 2022).

In summary, all these studies indicated that the gut microbiota is associated with epilepsy and therapy, but the causal mechanism of these associations has not yet been determined. However, these studies had several limitations. First, all these studies had small sample sizes, which impacted the results and conclusions. Second, the follow-up time of the studies was short. Third, most of the patients enrolled in the studies had taken antiseizure drugs, which could also influence the gut microbiota (Gong et al., 2022; Ilhan et al., 2022). Fourth, not all these studies controlled for confounding factors, such as diet, antibiotic discontinuation time, or age, which could also influence the alteration of the gut microbiota. Fifth, these studies did not draw the same conclusion about the exact bacterial effect on epileptogenesis. However, all these studies demonstrated a relationship between the gut microbiota and epilepsy. The human gut also contains fungi, viruses, parasites, and archaea; however, there were no studies that investigated their role in epilepsy. Therefore, future investigations are needed to better understand the role of these microbiota in epilepsy.

## 3 Methods for identifying the microbiota in humans

With the development of methods for identifying the microbiota, we can obtain more information about the gut microbiota and its metabolites. Next-generation sequencing (NGS) is the most used method for identifying the microbiota in humans. The key NGS methods include 16S ribosomal RNA (16S rRNA) and shotgun metagenomic sequencing. 16S rRNA sequencing is the most used method for bacterial identification. It is targeted for polymerase chain reaction amplification of the bacterial 16S ribosomal RNA (rRNA) gene and cannot detect fungi, parasites, or viruses (Durazzi et al., 2021). Moreover, 16S rRNA has a lower risk of host contamination (Liu et al., 2021). However, 16S rRNA sequencing has less sensitivity and cannot detect strain-level changes. In addition, 16S rRNA sequencing cannot provide a direct functional profile because the method only characterizes sequences from one essential gene. Shotgun metagenomics is more expensive than 16S rRNA sequencing but offers far broader species- and strain-level resolution, more accurate functional profiling, and possibly the identification of unknown species and strains of microbes (Jovel et al., 2016).

Sequencing platforms have also been developed that are classified by maximum output, reads per run, accuracy, run time, number of nucleic acids, and read length (Illumina, Ion Torrent, Sequencing by Oligonucleotide Ligation and Detection (SOLiD), PacBio, and Nanopore). Illumina sequencing technology is a method that allows for the simultaneous sequencing of millions of fragments and is a significant advancement over DNA sequencing due to its speed and cost (Bentley et al., 2008). Ion Torrent sequencing technology uses semiconductor chips to detect hydrogen ions released during DNA polymerization but is not widely used for detecting gut microbiota (Guerra et al., 2018). The SOLiD system has high-throughput sequencing capabilities and accuracy, particularly in the context of identifying single-nucleotide polymorphisms (SNPs) and other genetic variations, and can generate large amounts of data and detect both sequence and structural variations in the genome (McKernan et al., 2009). PacBio and nanopores can enable the sequencing of much longer DNA fragments than short-read sequencing platforms such as Illumina. These sequencing platforms improved the resolution of complex genomic regions, structural variations, and repetitive sequences. Nanopores can test nucleic acids, including both native DNA and RNA molecules, and have the advantages of rapid readout, high accuracy, low cost, and portability (Dorey and Howorka, 2024). It has been reported that nanopore sequences are promising for polypeptide identification and sequencing, capturing folded proteins, and achieving peptide threading (Dorey and Howorka, 2024). With the progress of sequencing technologies, we will learn more about the gut microbiota and epilepsy.

## 4 Antiseizure medications and the gut microbiota

Currently, an increasing number of studies have shown that antiseizure medications probably influence the gut microbiota, which will confound our understanding of the relationship between the gut microbiota and epilepsy. Gong et al. reported that valproate could increase the ratio of the phyla *Firmicutes* to *Bacteriodetes* in children with focal epilepsy (Gong et al., 2022). Ilhan et al. reported that antiseizure medications (carbamazepine, lamotrigine, and topiramate) reduced the growth of more than 10 bacterial strains, and antiseizure medications with syrup excipient and artificial sweeteners reduced or stimulated microbiota growth in HT-29 cells, but they did not report the effects of the patients’ symptoms (Ilhan et al., 2022). In addition, the gut microbiota can also affect the efficacy of drugs. *Akkermansia muciniphila* likely reduces the expression of drug-resistance genes in HT-29 cells, and *Bifidobacterium longum* may reduce the cytotoxic effects of carbamazepine and lamotrigine in cells (Ilhan et al., 2022). Thai et al. reported that topiramate significantly increased *Lactobacillus johnsonii* in naïve mice (Ilhan et al., 2022). Valproate prevents peritoneal adhesion following abdominal injury through chymase inhibition and decreases intestinal inflammation in individuals with IBS (Liu et al., 2020; Felice et al., 2021). Phenobarbital treats postnatal hyperbilirubinemia through its effects on the hepatic enzymatic elimination of bilirubin (Chawla and Parmar, 2010). Wan et al. also reported that non-responsive children with infantile epileptic spasm syndrome had upregulated *Clostridioides* and *Peptoclostridium*_phage_p630P2 and downregulated *Lachnospiraceae* and *Phascolarctobacterium* after adrenocorticotropic hormone (ACTH) treatment (Fairlie et al., 2020). In summary, these antiseizure medications were only found to affect the gut microbiota. It is important to note that no research has yet explored the potential correlation between the efficacy of antiseizure medications and alterations in gut microbiota. Additional studies have confirmed that alterations in the gut microbiota can be influenced by antiseizure medications and their ingredients.

## 5 The mechanisms through which the gut microbiota interacts with the brain

### 5.1 Gut microbiota-mediated metabolites

The gut-brain signaling pathways primarily rely on vagal receptors located in the gastrointestinal mucosa, which are capable of detecting changes in the gut microbiota and associated metabolites and transmitting these signals to the CNS (de Lartigue et al., 2011). Various factors, such as gut peptides, inflammatory molecules, and food or drug components, can influence the composition of gut microbiota and its metabolites (Sen et al., 2017). Metabolomics has revealed that microbiota-mediated metabolites, including short-chain fatty acids (SCFAs), phenolic compounds, and free amino acids, are implicated in epilepsy (Brandsma et al., 2019; Boeri et al., 2022). Dietary fibers are fermented and subsequently produce SCFAs by some gut microbiota, including *Akkermansia municiphilla*, *Ruminococcus bromii*, and *Faecalibacterium prausnitzii* (Morrison and Preston, 2016). Circulating SCFAs can cross the blood–brain barrier (BBB) as signaling molecules within the gut–brain axis. They directly or indirectly provide energy to neurons while maintaining glucose and energy homeostasis and regulate neurotransmitters’ function (Hu et al., 2018). Notably, drug-resistant epilepsy often coincides with energy deficiency (Araujo et al., 2014), suggesting that SCFAs might play an important role in the occurrence and progression of epilepsy. Importantly, a KD, which is a treatment for drug-resistant epilepsy, can reduce seizure frequency, probably through the production of SCFAs (Gong et al., 2021).

Phenolic compounds produced by the gut microbiota principally consist of serotonin, dopamine, γ-aminobutyric acid (GABA), and norepinephrine (NE) (Maia et al., 2017; Uchida et al., 2017). Furthermore, elevated endogenous noradrenergic transmission causes an increase in NE release, which is involved in some cases of epilepsy (Fitzgerald, 2010).

The relationship between free amino acids (FAAs) and the gut microbiota is also bidirectional (Kumar et al., 2018). Through metabolomic sequencing, Zhou et al. discovered that tryptophan and kynurenine levels were decreased in adults with epilepsy, suggesting their potential as biomarkers for epilepsy. Additionally, they observed positive associations between *Providencia, Candidatus Marithrix, Ulvibacter, Methanospirillum*, and *Gaetbulibacter* and serum tryptophan levels. Furthermore, *Providencia, Mesotoga*, and *Desulfurispora* exhibited positive correlations with serum kynurenine (Zhou et al., 2023). It has been reported that tryptophan can be metabolized to kynurenine, which regulates neuroendocrine and intestinal immune effects (Gao et al., 2020). Moreover, high concentrations of kynurenine have been shown to possess neuroprotective effects and regulate glutamate function (Rho, 2004). Few studies have explored the relationship between epilepsy and FAAs. This could be a future research direction.

### 5.2 Immune system pathways

The gut communicates with the brain through immunological pathways. The gut microbiota can influence the development and function of microglia, which are crucial resident immune cells in the CNS involved in CNS disorders (Maneu et al., 2016). Microglia activation occurs during epileptogenesis in both rat and human brains affected by epilepsy (Yankam et al., 2017). Probiotics reduce microglia activation in the hippocampus via the vagus nerve through the microbiota–gut brain axis (Liu et al., 2021). In the mouse, in the case of hepatitis virus-induced nerve injury, the gut microbiota can enhance the ability of microglia to respond to infection and prevent neurological diseases (Brown et al., 2019). *B. longum* can mediate tryptophan metabolism to ameliorate microglial activity in the cerebellum (Brown et al., 2019). Microglia are activated by microbial metabolites, including cytokines and hormones, and inhibited by SCFAs and tryptophan derivatives (Zheng et al., 2023). Braniste et al. reported that abnormal gut microbiota in mice is directly associated with reduced expression of tight junction proteins, including occludin and claudin-5, leading to an increase in BBB permeability (Braniste et al., 2014). During systemic inflammation, increases in the levels of inflammatory cytokines, such as interleukin-1β (IL-1β), interleukin-6 (IL-6), and tumor necrosis factor-α (TNF-α), can accelerate the disruption of the BBB (Geng et al., 2018; Kim et al., 2022). In patients with temporal lobe epilepsy, chronic inflammation is very common in brain parenchyma (Gales and Prayson, 2017) and may lead to mitochondrial dysfunction (Volmering et al., 2016).

## 6 Potential treatments for patients with refractory epilepsy associated with the microbiota

To date, there are no completely effective or safe therapies for refractory epilepsy. However, a systematic review reported that manipulating the gut microbiota through the use of a fecal microbiota transplant (FMT), antibiotic treatment, a ketogenic diet, or prebiotic treatment could improve dysbiosis and ameliorate epilepsy-induced brain injury to some extent (Arulsamy et al., 2020). Thus, in this section, the important potential treatments for epilepsy are summarized.

### 6.1 Ketogenic diet

A KD contains a high fat content, low carbohydrate content, appropriate protein levels, and other nutrients (Rekdal and Balskus, 2018). Many recent studies have shown that a KD has a positive effect on many disorders, such as Alzheimer’s disease (Lim et al., 2022), malignant tumors (Cohen et al., 2018; Ok et al., 2018; Iyikesici, 2019), obesity (Moreno et al., 2016; Yuan et al., 2022), diabetes (Saslow et al., 2017), and epilepsy (Cohen et al., 2018; Fan et al., 2019; Iyikesici, 2019). Recently, several studies have shown that the abundances of *Akkermansia* and *Parabacteroides* increase after KD treatment (Newell et al., 2016; Xie et al., 2017; Olson et al., 2018; Lindefeldt et al., 2019). Previous studies have shown that the beneficial effects of *Akkermansia muciniphila* are associated with SCFAs following KD consumption (Paoli et al., 2019; Attaye et al., 2021). Additionally, the abundance of *Bifidobacteria* decreased after KD treatment, and these bacteria displayed antiseptic effects (Lindefeldt et al., 2019; Dahlin et al., 2022). Some studies have shown that α-diversity decreases, the abundance of the phylum *Bacteroidetes* increases, and the abundances of the phyla *Proteobacteria* and *Firmicutes* decrease in children with refractory epilepsy after KD treatment (Xie et al., 2017; Zhang et al., 2018). Thus, scientists speculate that the antiepileptic effect of KD therapy might depend on the gut microbiota. As shown by Olson et al., germ-free mice with epilepsy are resistant to KD treatment, while epileptic mice treated with *Akkermansia* and *Parabacteroides* exhibit a satisfactory response (Olson et al., 2018). This study confirmed that the gut microbiota is an essential link for the antiepileptic effect of a KD. In summary, a KD is a traditional but effective therapy for epilepsy and most likely works through alteration of the gut microbiota.

### 6.2 Probiotics and prebiotics

Probiotics are beneficial to host health when they are supplied appropriately. These bacteria mainly consist of *Lactobacillus*, *Bifidobacterium*, *Lactococcus*, *Saccharomycetes*, and several enzymes. Many researchers have reported that probiotics can prevent or treat many disorders, such as allergies and infectious diseases (Ishaque et al., 2018; Trick et al., 2018). Because of the relationship between the gut microbiota and the brain, probiotics are used to prevent and treat CNS-related diseases. For example, treatment with *L. plantarum MTCC1325* can significantly improve cognition, ameliorate brain acetylcholine levels, and resolve histopathological lesions in albino rats with D-galactose-induced Alzheimer’s disease (Nimgampalle and Kuna, 2017). A recent observational study showed that, compared with healthy controls, newborns with rotaviral infection are more likely to suffer from neonatal seizures and that treatment with probiotics immediately after birth can reduce this risk (Yeom et al., 2019). Additionally, the use of probiotics as a supplementary treatment can obviously improve seizure control and quality of life in patients with drug-resistant epilepsy (Gomez-Eguilaz et al., 2018). Oral probiotic treatment dramatically reduces seizure severity and improves spatial learning and memory in Pentylenetetrazol-induced kindling in rats (Bagheri et al., 2019). These results indicate that probiotic therapy is promising for the prevention and treatment of epilepsy. Prebiotics are non-digestible food ingredients that selectively stimulate the growth and activity of one or a few gut microbes, such as *Bifidobacteria* and *Lactobacillus*, and improve the intestinal microenvironment (Chen and Quigley, 2014). A recent systematic review and meta-analysis of randomized controlled trials (RCTs) confirmed that prebiotics can increase the abundance of *Bifidobacteria* in patients with IBS or other functional bowel disorders (Wilson et al., 2019). Prebiotics are also effective for treating several diseases of the CNS. For children with ASD, prebiotic intervention can significantly improve psychological traits (Grimaldi et al., 2018). An RCT by Barichella et al. showed that fermented milk containing multiple probiotics and prebiotic fiber can improve constipation in patients with Parkinson’s disease (Barichella et al., 2016). However, although the gut microbiota is closely related to epilepsy, no studies have focused on the effect of prebiotics on this disease. This might be a research direction in the future.

### 6.3 Fecal microbiota transplant

Fecal microbiota transplant (FMT) is a therapeutic approach aimed at rebuilding the gut microbiota. By transferring an intestinal microbiota obtained from the feces of a healthy donor into the patient’s gastrointestinal tract, FMT has proven to be an efficacious treatment for recurrent *Clostridium difficile* infection (Kelly et al., 2016; Hvas et al., 2019). FMT is also used for the treatment of inflammatory bowel disease or irritable bowel syndrome and can ameliorate the depression and anxiety symptoms induced by IBS (Kurokawa et al., 2018). Only two studies have evaluated FMT for the treatment of epilepsy. He et al. studied a 22-year-old girl with Crohn’s disease who had a history of epilepsy for 17 years and who received FMT treatment. Following the FMT, she was seizure-free without antiseizure medication for 20 months and experienced remission of intestinal symptoms (He et al., 2017), indicating that FMT has good prospects for epilepsy treatment. However, the safety of FMT needs to be further explored. DeFilipp et al. reported that two patients suffered from severe extended-spectrum beta-lactamase (ESBL)-producing *Escherichia coli* bacteremia after FMT, and one patient died from this bacteremia (DeFilipp et al., 2019). In both cases, the bacteremia was caused by ESBL-producing *Escherichia coli* from the donor feces, and it was subsequently transmitted through FMT. Identifying healthy and eligible donors is essential (Kassam et al., 2019).

We can easily identify gut microbes and mechanisms in animal models; however, it is difficult to translate these findings to humans. In humans, observational studies have shown differences in the gut microbiota between patients and healthy people, but causal relationships have not been assessed. Furthermore, the findings regarding gut microbiota in various studies have demonstrated inconsistencies due to variations in subjects’ ages, races, and dietary habits. In addition, Dahlin et al. found that *Bifidobacteria* decreased after KD treatment; however, Zhang et al. reported that *Firmicutes* and *Actinobacteria* both decreased after KD therapy and *Bacteroidetes* increased after KD therapy (Zhang et al., 2018; Dahlin et al., 2022). The alterations in the gut microbiota in different studies are displayed in Table 1. The composition of the gut microbiota was assessed using next-generation sequencing of 16S ribosomal RNA genes or whole-genome shotgun sequencing based on DNA-associated methods, which also allows the inference of microbiota functions (Ranjan et al., 2016; Vogtmann et al., 2016). Recently, metabonomics has been used to identify metabolic products of the gut microbiota in stool and serum, which provides hope for the future study of the gut microbiota (Zhao et al., 2017).

**Table 1:** Focus on the gut microbiota and epilepsy in human studies.

Epilepsy group and etiology	Heath group	Seizure type	Treatment and duration	Results	Samples	Methods	References
*N* = 14, pediatric patients with refractory epilepsy Age: 10 m to 3y4m Etiology: no known genetic metabolic disorders or severe systemic illnesses	*N* = 30 Age 1y1m to 2y10m	/	A ketogenic diet for a week	↓ Diversity in infants with epilepsy ↑ The phylum Firmicutes and the genus *Cronobacter* in infants with epilepsy ↓ *Prevotella* and *Bifidobacterium* in infants with epilepsy ↑ The phylum Proteobacteria in infants with refractory epilepsy ↑ The phylum Bacteroidetes after KD treatment ↓ The phylum Proteobacteria after KD therapy - The phylum Firmicutes did not change after KD therapy	Fecal samples	16S rRNA sequencing	Xie et al. (2017)
*N* = 42, drug-resistant epilepsy patients Age: 28.4 ± 12.4 years *N* = 49, drug-sensitive epilepsy patients Age: 25.1 ± 14.6 years Etiology: NA	*N* = 65 (from the same families of the patients and had the same eating habits) Age: 29.4 ± 13.8 years	Generalized, Focal, Multiple forms	/	↑ α-diversity in patients with DRE ↑ The phyla Firmicutes and Verrucomicrobia in patients with DRE ↑ Bifidobacteria and Lactobacilli in patients who experienced four or fewer seizures per year compared to more seizures per year	Fecal samples	16S rDNA sequencing	Peng et al. (2018)
*N* = 20 Age: 2.8–15.3 years Etiology: unknown, genetic, and structural	/	Focal, generalized tonic–clonic seizure, myoclonic, atypical absent seizure	The ketogenic diet for 6 months	↓ α-diversity after KD therapy ↓ *Firmicutes* and *Actinobacteria* after KD therapy ↑ *Bacteroidetes* after KD therapy	Fecal samples	16S rDNA sequencing	Zhang et al. (2018)
*N* = 12 Age: 2.8–15.3 years Etiology: unknown, genetic, and structural	*N* = 11	Tonic, generalized tonic–clonic, focal, myoclonic-atonic, epileptic spasms, atypical absences, continuous spike–wave during sleep	The ketogenic diet for 3 months	- α-diversity no change after KD treatment ↓ *Bifidobacteria*, *E. rectale, Dialister,* and *E. coli* after KD treatment	Fecal samples	16S rRNA Sequencing	Lindefeldt et al. (2019)
*N* = 25 pediatric patients with cerebral palsy and epilepsy Age: 108.13 ± 42.83 months.	*N* = 12 Age: 70.43 ± 20.93	/	/	↑ Diversity ↑ Genera *Bifidobacterium, Streptococcus, Akkermansia, Enterococcus, Prevotella, Veillonella, Rothia, and Clostridium IV* ↓ Genera *Bacteroides, Faecalibacterium, Blautia, Ruminococcus, Roseburia, Anaerostipes, and Parasutterella*	Fecal samples	16S rRNA sequencing	Huang et al. (2019)
*N* = 55, drug-resistant epilepsy (30) and drug-sensitive epilepsy (25) Age: 26.33 ± 12.05 years Etiology: unknown causes	*N* = 46 Age: 28.5 ± 4.27 years	Focal onset, Generalized onset, Unknown onset	/	↓ α-diversity in epileptic patients ↑ Actinobacteria and Verrucomicrobia at the phylum level, *Prevotella_9, Blautia,* and *Bifidobacterium* at the genus level in epileptic patients	Fecal samples	16S rRNA sequencing	Gong et al. (2020)
				↓ Proteobacteria at the phylum level in epileptic patients DRE: ↑ Actinobacteria, Verrucomicrobia, and Nitrospirae at the phylum level, and *Blautia*, *Bifidobacterium*, *Subdoligranulum*, *Dialister*, and *Anaerostipes* at the genus level in patients with drug-resistant epilepsy			
*N* = 30, idiopathic focal epilepsy Age: 41.3 ± 12.2 years Etiology: unknown causes	*N* = 10 Age: 31.7 ± 6.8 years	/	/	↑ genera of *Campylobacter*, *Delftia*, *Haemophilus*, *Lautropia*, and *Neisseria* among the Proteobacteria phylum in patients with epilepsy The Fusobacteria phylum only detected in patients with epilepsy	Fecal samples	16S rDNA sequencing	Safak et al. (2020)
*N* = 8 Age: 1–7 years Etiology: unknown, genetic, and structural	*N* = 32 Age: 1–7 years	Infantile spasms, Generalized, Focal and generalized, Focal	/	↑ Actinobacteria ↓ Bacteroidetes and Proteobacteria in DRE *Enterococcus faecium* group, *Bifidobacterium longum* group, and *Eggerthella lenta* are probably biomarkers for intractable epilepsy	Fecal samples	16S rRNA Sequencing	Lee et al. (2020)
*N* = 25 Age: 30.21 ± 15.53 years Etiology: NA	*N* = 50 Age: 30.1 ± 5.72 years	Generalized (17), simple partial seizure (3), complex partial seizure (4)	/	↓ α-diversity in epileptic patients ↑ Proteobacteria and Actinobacteriota in phylum in epilepsy patients ↓ *Bacteroidota* in phylum in epilepsy patients ↑ *Faecalibacterium*, *Escherichia-Shigella*, *Subdoligranulum*, and *Enterobacteriaceae-unclassified* in genera in epilepsy patients ↓ *Bacteroides, Megamonas, Prevotella, Lachnospiraceae-unclassified,* and *Blautia* in genera in epilepsy patients	Fecal samples	16S rRNA sequencing	Cui et al. (2021)
*N* = 44, drug-responsive and drug-resistant groups Age: 1–7 years Etiology: unknown, genetic, and structural			/	↑ *Bacteroides finegoldii* and *Ruminococcus_g2* in DSE ↑ *Negativicutes* (belong to Firmicutes) in DRE	Fecal samples	*/*	Lee et al. (2021)
*N* = 29 Age: 3–13 months Etiology: unknown (20), TSC (3), FCD (1), NF1 (1), congenital cleft malformation (1) and HIE (3)	*N* = 29 Age: 3–13 months	Epileptic spasms	ACTH for 2 weeks	↓ Abundance of the genus *Akkermansia* after ACTH treatment	Fecal samples	16S rRNA sequencing	Xu et al. (2021)
*N* = 10 Age: 6 years (5, 9) years	*N* = 14 Age: 5 years (4, 8) years	Focal	Oxcarbazepine for 3 months	Before treatment ↑ At the phylum level: Actinobacteria ↑ At the genus level: *Escherichia/Shigella, Streptococcus, Collinsella,* and *Megamonas* After the treatment ↓ At the genus level: *Escherichia/Shigella, Streptococcus, Collinsella*, and *Megamonas*	Fecal samples	16S rDNA sequencing	Gong et al. (2022)
*N* = 10 drug-resistant epilepsy patients Age: 27.70 ± 13.32 years *N* = 10 non-drug-resistant epilepsy patients Age: 23.20 ± 11.25 years	*N* = 19 Age: 25.47 ± 14.03	Focal, Generalized, Combined Generalized and Focal	/	↑ the phylum *Firmicutes* and the class Bacilli in patients with drug-resistant epilepsy ↑ *Bacteroidetes* in the non-epileptic group ↑ *Proteobacteria* and *Actinobacteria* in the epilepsy group	Fecal samples	16S rDNA sequencing	Dai et al. (2022)
*N* = 10 Age: 53 ± 6.72 years Etiology: Unknown	/	Focal, generalized, unknown	VPA (1,000 mg) daily for 3 months	No changes in gut microbiota richness and complexity ↑ The ratio of phyla *Firmicutes* to *Bacteriodetes* after 3 months of VPA-treatment	Fecal samples	16S rRNA sequencing	Gong et al. (2022)
*N* = 10 Age: 6.35 years (5.40, 9.50) years	*N* = 10 Age: 5.15 years (3.98, 7.90) years		Oxcarbazepine (20 mg/kg/d to 30 mg/kg/d) for 3 months	Pretreatment (epilepsy group vs. control group) ↑ At the phyla level, Actinobacteria ↑ At the genus level, *Escherichia/Shigella*, *Streptococcus*, *Collinsella*, and *Megamonas* ↓ *Faecalibacterium*, *Escherichia*/*Shigella*, and *Collinsella* ↓ Simpson index Posttreatment (epilepsy group vs. pretreatment) ↓ at the phyla level, Actinobacteria ↓ At the genus level, *Escherichia/Shigella* and *Streptococcus*	Fecal samples	16S rDNA sequencing	Zhou et al. (2022)
A bidirectional Mendelian randomization study		Absence, generalized epilepsy with tonic–clonic seizures, juvenile myoclonic	/	↑ Veillonellaceae increase the risk of childhood absence epilepsy ↓ Class Melainabacteria decrease the risk of generalized epilepsy with tonic–clonic seizures ↓ Class Betaproteobacteria and order Burkholderiales decrease the risk of juvenile myoclonic epilepsy	/	/	Ouyang et al. (2022)
A 17-year-old girl Etiology: mutations in the PGM1 and EEF1A2 genes	/	Generalized seizures	A ketogenic diet followed by a low fermentable oligosaccharides, disaccharides,	↓ *Firmicutes, Bacteroidetes,* and *Proteobacteria* after KD treatment *↓* Actinobacteria*, Firmicutes, Lactobacilli,* and *Bifidobacteria* after a low FODMAP diet	/	/	Bertuccioli et al. (2022)
			monosaccharides, and polyols (FODMAP) diet				
*N* = 27 children with cerebral palsy and epilepsy Age: 4–14 years	/	/	/	In the oral cavity (vs. control group): ↓ Firmicutes and Bacteroides ↑ Actinomycetes In gut microbiota (vs. control group): *Bifidobacterium*, *Bacteroidetes*, and *Prevotella* were the top three abundant genera	Fecal samples	16S rDNA sequencing	Huang et al. (2022)
*N* = 28, children with drug-resistant epilepsy Age: 1.5–18 years Etiology: structural, genetic, and unknown	/	Focal, epileptic spasms, tonic, myoclonic, generalized tonic–clonic	The ketogenic diet for 3 months	↓ Bifidobacteria after KD treatment	Fecal samples	16S rDNA sequencing	Dahlin et al. (2022)
*N* = 22 Age: 18–60 years Etiology: unknown (12), genetic (9) and structural (9)	*N* = 10	Focal generalized	/	↑ Actinobacteria, Bacteroidetes, and Proteobacteria at the phylum level in patients with epilepsy ↓ Firmicutes at the phylum level in patients with epilepsy	Fecal samples	16S rDNA sequencing	Zhou et al. (2023)
*N* = 20 Age: 2–11 years Etiology: Unknown	*N* = 7	/	/	↑ The genera *Flavobacterium, Holdemania, and Hyphomicrobium* The genera *Megamonas* and *Coriobacterium* were observed only in patients with epilepsy	Fecal samples	16S rRNA sequencing	Turay et al. (2023)
*N* = 13 children with cerebral palsy with epilepsy Age: 1–16 years old	*N* = children with non-epileptic cerebral palsy	/	/	*↓ Bacteroides fragilis* and *Dialister invisus* vs. children with non-epileptic cerebral palsy ↑ *Phascolarctobacterium faecium* and *Eubacterium limosum* vs. children with non-epileptic cerebral palsy	Fecal samples	Shotgun metagenomic sequencing	Peng et al. (2023)
*N* = 30 Age: 4.8 months (1.75, 10) months Etiology: unknown (12), genetic (9), and structural (9)	/	Epileptic spasms	ACTH for 14 days	- α-diversity no change after ACTH treatment ↑ *Clostridioides* and *Peptoclostridium*_phage_p630P2 in the non-responsive group after ACTH treatment ↓ *Lsenella* and *Phascolarctobacterium* in the non-responsive group after ACTH treatment	Fecal samples	16S rRNA sequencing	Wan et al. (2024)

FCD, focal cortical dysplasia; HIE, Hypoxic–ischemic encephalopathy; NF1, neurofibromatosis 1; TSC, tuberous sclerosis; DRE, drug-resistant epilepsy; DSE, drug-sensitive epilepsy.

## 7 Conclusion

In this review, we present a summary of human studies investigating the relationship between gut microbiota and epilepsy and discuss several potential therapeutic approaches for modulating gut microbiota in individuals with epilepsy, including KD, FMT, and probiotics or prebiotics interventions. The relationship between gut microbiota and epilepsy is probably related to heritability. Zhernakova et al. reported that host genetics regulate the genetic diversity of gut microbiota (Zhernakova et al., 2024). Furthermore, a bidirectional Mendelian randomization study demonstrated that the composition of the gut microbiota is associated with seizure type; for example, the family *Veillonellaceae* is associated with a greater risk of childhood absence epilepsy, the class *Melainabacteria* is associated with a lower risk of generalized epilepsy with tonic–clonic seizures, and the class *Betaproteobacteria* and the order *Burkholderiales* are associated with a lower risk of juvenile myoclonic epilepsy. These findings indicated that genetic factors or seizure types are likely involved in the relationship between the gut microbiota and epilepsy. Therefore, the alteration of the gut microbiota is probably the result of the individual genetic background and environment. Future studies should focus on the etiology of epilepsy and identifying genetic mutations associated with gut microbiota in order to expand our understanding of these gene functions. With advancements in genomics technology, elucidating both associations and causal relationships will undoubtedly unravel the intricate interplay between gut microbiota and epilepsy while simultaneously identifying novel drug targets from a microbial perspective.

## Author contributions

QL: Conceptualization, Writing – original draft. YG: Writing – original draft. JL: Writing – review & editing. ZY: Writing – review & editing. JQ: Writing – review & editing.
